# Immunosuppressive effect of hispidulin in allergic contact dermatitis

**DOI:** 10.1186/s12906-019-2689-z

**Published:** 2019-10-15

**Authors:** Premrutai Thitilertdecha, Panwadee Pluangnooch, Sunita Timalsena, Kitipong Soontrapa

**Affiliations:** 1 Siriraj Research Group in Immunobiology and Therapeutic Sciences, Faculty of Medicine Siriraj Hospital, Mahidol University, Bangkok, Thailand; 2 Department of Pharmacology, Faculty of Medicine Siriraj Hospital, Mahidol University, 2 Wanglang Road, Bangkoknoi, Bangkok, 10700 Thailand

**Keywords:** Hispidulin, Immunosuppressive drug, Contact hypersensitivity, Atopic dermatitis, T cells, 1-fluoro-2,4-dinitrobenzene

## Abstract

**Background:**

Long-term use of most immunosuppressants to treat allergic contact dermatitis (ACD) generates unavoidable severe side effects, warranting discovery or development of new immunosuppressants with good efficacy and low toxicity is urgently needed to treat this condition. Hispidulin, a flavonoid compound that can be delivered topically due to its favorable skin penetrability properties, has recently been reported to possess anti-inflammatory and immunosuppressive properties. However, no studies have investigated the effect of hispidulin on Th1 cell activities in an ACD setting.

**Methods:**

A contact hypersensitivity (CHS) mouse model was designed to simulate human ACD. The immunosuppressive effect of hispidulin was investigated via ear thickness, histologic changes (i.e., edema and spongiosis), and interferon-gamma (IFN-γ) gene expression in 1-fluoro-2,4-dinitrobenzene (DNFB)-sensitized mice. Cytotoxicity, total number of CD4^+^ T cells, and percentage of IFN-γ-producing CD4^+^ T cells were also investigated in vitro using isolated CD4^+^ T cells from murine spleens.

**Results:**

Topically applied hispidulin effectively inhibited ear swelling (as measured by reduction in ear thickness), and reduced spongiosis, IFN-γ gene expression, and the number of infiltrated immune cells. The inhibitory effect of hispidulin was observed within 6 h after the challenge, and the observed effects were similar to those effectuated after dexamethasone administration. Hispidulin at a concentration up to 50 μM also suppressed IFN-γ-producing CD4^+^ T cells in a dose-dependent manner without inducing cell death, and without a change in total frequencies of CD4^+^ T cells among different concentration groups.

**Conclusion:**

The results of this study, therefore, suggest hispidulin as a novel compound for the treatment of ACD via the suppression of IFN-γ production in Th1 cells.

## Background

Allergic contact dermatitis (ACD), also known as contact hypersensitivity (CHS), is a type 1 T helper (Th1) cell-mediated inflammatory skin disease caused by repeated topical exposure to a previously sensitized. A CHS mouse model has been utilized in studies on the pathophysiology of ACD [1–3]. For treatment of ACD, topical and systemic administrations of immunosuppressive drugs, such as corticosteroids, cyclosporine-A and tacrolimus, are normally used. However, these drugs associated with various adverse effects after prolonged use [4–6], so it is of importance to find a novel candidate compound to treat ACD. Recently, phytochemicals have become good candidate active compounds for drug discovery and for the development of novel immunosuppressive treatments for ACD due to their high efficacy and low toxicity.

Hispidulin (4′,5,7-trihydroxy-6-methoxyflavone), a flavonoid compound found in several plants [7–13], has recently been investigated for its anti-inflammatory and immunosuppressive ability to treat several inflammatory and autoimmune diseases. Previously, a number of reports showed that hispidulin suppressed inflammations in mouse models of 12-O-tetradecanoylphorbol-13-acetate (TPA)-induced ear edema [9, 10], croton oil-induced dermatitis [12], ultraviolet A radiation-induced skin damage [14], and passive cutaneous anaphylaxis [15]. At the cellular level, hispidulin could inhibit functions of various immune cells like T cells [13], macrophages [10] and mast cells [15]. Its anti-inflammatory activity was reported to be mediated via nuclear factor erythroid 2-related factor 2 (Nrf2)/heme oxygenase (HO) -1 signaling [10], not nuclear factor kappa-light-chain-enhancer of activated B cells (NF-κB) induction [11], and downregulation of inducible nitric oxide synthase (iNOS) and cyclooxygenase (COX)-2 expressions [8]. Although many anti-inflammatory and immunosuppressive properties of hispidulin have been appreciated, the effect of hispidulin on ACD via Th1 cell activities has not been explored yet. Accordingly, the aim of this study was to investigate the immunosuppressive effect of purified hispidulin on Th1 cell function in the DNFB-induced CHS mouse model.

## Materials and methods

### Animals

Male C57BL/6 mice, 7–10 weeks of age, were obtained from the National Laboratory Animal Center, Mahidol University, Salaya Campus, Nakhon Pathom, Thailand. All mice were housed in a 12-h/12-h light/dark specific pathogen-free condition, with free access to standard rodent feed and water. The care and treatment of study mice was in accordance with the guidelines of Mahidol University and the Office of the National Research Council of Thailand (NRCT). The total number of animals used in each experimental group was referred from the published article conducting the similar experiments [16]. All experimental protocols were approved by the Siriraj Animal Care and Use Committee (Si-ACUC), Faculty of Medicine Siriraj Hospital, Mahidol University, Bangkok, Thailand (COA no. 015/2559).

### Chemicals and reagents

Fluorescein isothiocyanate (FITC) conjugated antibody to mouse CD4, and purified antibodies to mouse-CD3, −IL-4, and -CD28 were purchased from eBioscience, Inc. (San Diego, CA, USA). Phycoerythrin (PE) conjugated antibody to mouse-IFN-γ, and recombinant mouse (rm) IL-12 were purchased from BioLegend, Inc. (San Diego, CA, USA). Recombinant mouse IL-2 was purchased from ImmunoTools GmbH (Friesoythe, Germany). 1-Fluoro-2,4-dinitrobenzene (DNFB), hispidulin (purity > 98%), dexamethasone, phorbol 12-myristate 13-acetate (PMA), and ionomycin were purchased from Sigma-Aldrich Corporation (St. Louis, MO, USA). Paraformaldehyde was purchased from Merck (Darmstadt, Germany). GolgiPlug™ Protein Transport Inhibitor and Cytofix/Cytoperm™ Fixation/Permeabilization Solution Kit were purchased from BD Biosciences (Franklin Lakes, NJ, USA). Propidium iodide (PI) solution was purchased from Miltenyi Biotec (Bergisch Gladbach, Germany). Phosphate buffered saline (PBS) was purchased from AMERESCO, Inc. (Framingham, MA, USA. Roswell Park Memorial Institute (RPMI)-1640 Complete Medium (Gibco; Thermo Fisher Scientific, Waltham, MA, USA), fetal bovine serum (FBS) (Biochrom; Merck, Darmstadt, Germany), olive oil (ChemCruz™; Santa Cruz Biotechnology, Inc., Dallas, TX, USA), acetone (Scharlau; Scharlab, S.L., Barcelona, Spain), and thiopental sodium (THIOPEN; Unique Pharmaceutical Laboratories Ltd., Ankleshwar, India) were also used.

### Induction of contact hypersensitivity (CHS)

Twenty-five mice were sensitized by painting 25 μL of 0.5% DNFB in a mixture solution of acetone and olive oil (4:1, v/v) on the shaved abdomen (day 0) with the average area of 2 × 2 cm. Five days after sensitization, mice were elicited by painting 20 μL of 0.3% DNFB in the same vehicle on the dorsal and ventral pinna of both ears. For the topical application of drug compounds, mouse pinna was applied with either hispidulin (10 or 30 μg/ear, treatment groups, *n* = 5 each concentration), dexamethasone (30 μg/ear, positive control, *n* = 5), or no treatment (the vehicle alone, a sensitized control, *n* = 5) starting on day 4 for 3 consecutive days. Mice sensitized and elicited with vehicle alone were used as a non-sensitized control (*n* = 5). Ear thickness of each ear of each individual mouse was measured at pre-challenge and at 6-, 24-, and 48-h post-challenge using a Vernier caliper (Mitutoyo, Kanagawa, Japan).

### Histopathologic examination

At 48-h post-challenge, all mice were sacrificed by intraperitoneal injection of thiopental sodium (50 mg/kg). The mouse pinna was resected, fixed with 4% paraformaldehyde, and embedded in paraffin. Sections (5 μm thick) were stained with hematoxylin and eosin (H & E) for histopathologic evaluation of CHS.

### Measurement of IFN-γ gene expression

Total ribonucleic acid (RNA) was isolated from the excised pinna from one ear per mice at 24-h post-challenge using a Total RNA Extraction Kit (RBC Bioscience, New Taipei City, Taiwan). Complementary deoxyribonucleic acid (cDNA) was then synthesized from 1 μg of total RNA using an iScript™ Select cDNA Synthesis Kit (Bio-Rad Laboratories, Inc., Hercules, CA, USA). Real-time polymerase chain reaction (real-time PCR) analysis was performed using a LightCycler® 480 Instrument II (Roche Applied Science, Penzberg, Germany). The final reaction mixture in each reaction tube consisted of 0.5 μL of cDNA; 0.4 μL of sense and antisense primer solutions, including IFN-γ (forward: 5′-AACGCTACACACTGCATCT-3′, reverse: 5′-TGCTCATTGTAATGCTTGG-3′) and β-actin (ACTB, forward: 5′-ATGGATGACGATATCGCT-3′, reverse: 5′- ATGAGGTAGTCTGTCAGGT-3′) (Integrated DNA Technologies, Coralville, IA, USA); 10 μL of KAPA SYBR® Fast qPCR Master Mix (2x) Kit (Kapa Biosystems, Wilmington, MA, USA); and, 8.7 μL of sterile Milli-Q® water (Merck). The conditions for reverse transcription and the PCR steps were according to the manufacturer’s instructions. The normalization and quantification of mRNA expression was performed using LightCycler® 480 software (Roche Applied Science, Rotkreuz, Switzerland).

### In vitro testing for drug toxicity

Three naïve mice were sacrificed by intraperitoneal injection of thiopental sodium (50 mg/kg). Naïve CD4^+^ T cells were isolated from spleens by using CD4 MicroBeads and a MiniMACS™ Separator (Miltenyi Biotec) according to the manufacturer’s instructions. The isolated CD4^+^ T cells were then cultured at a cell density of 1 × 10^5^ cells/well in RPMI 1640 Complete Medium containing 10% FBS. Cells were also incubated in the absence or presence of hispidulin at different concentrations of 6.25 μM, 12.5 μM, 25 μM, and 50 μM for 7 days. After incubation, cells were stained with propidium iodide (PI) and analyzed using a CytoFLEX Flow Cytometer and CytExpert software (Beckman Coulter, Inc., Brea, California, US).

### Th1 cell differentiation assay and intracellular cytokine staining for IFN-γ production

Isolated naïve CD4^+^ T cells were obtained from spleens of four naïve mice using CD4 MicroBeads and a MiniMACS™ Separator (Miltenyi Biotec), and then cultured in RPMI 1640 Complete Medium containing 10% FBS. For Th1 cell differentiation, isolated CD4^+^ T cells were stimulated with anti-CD3 (10 μg/mL) and anti-CD28 (5 μg/mL) for 2 consecutive days. Activated cells were then cultured in Th1-skewing condition consisting of recombinant mouse (rm) IL-2 (2500 U/mL), rm. IL-12 (10 ng/mL), and anti-IL-4 (10 μg/mL), together with/without hispidulin (6.25 μM, 12.5 μM, 25 μM, and 50 μM) for 7 days prior to intracellular cytokine staining for IFN-γ production.

For intracellular cytokine staining, cultured CD4^+^ T cells at 1 × 10^6^ cells/mL were stimulated with 50 ng/mL PMA and 1 μg/mL ionomycin in the presence of GolgiPlug™ Protein Transport Inhibitor containing brefeldin A (BFA). Stimulated cells were then incubated at 37 °C in a 5% CO_2_ condition for 4 h before being surface stained with anti-mouse CD4 FITC at 4 °C for 30 min and then washed once. Stained cells were then fixed and permeabilized with 0.1 mL Cytofix/Cytoperm™ solution (BD Biosciences) at 4 °C for 20 min and washed with BD Perm/Wash™ Buffer (BD Biosciences) prior to centrifugation at 500 g for 5 min. Intracellular cytokine staining (ICS) was performed using anti-IFN-γ PE at 4 °C for 30 min before washing with BD Perm/Wash™ Buffer and resuspension in PBS. Stained samples were analyzed using a CytoFLEX Flow Cytometer and CytExpert software (Beckman Coulter).

### Statistical analysis

All data were analyzed using GraphPad Prism 7 software (GraphPad Software, San Diego, CA, USA), and are presented as mean ± standard deviation. One-way analysis of variance (ANOVA) followed by Bonferroni correction was used for multiple comparisons. Differences with a *p*-value less than 0.05 were considered statistically significant.

## Results

### Hispidulin-induced suppression of CHS responses

**Fig. 1:**
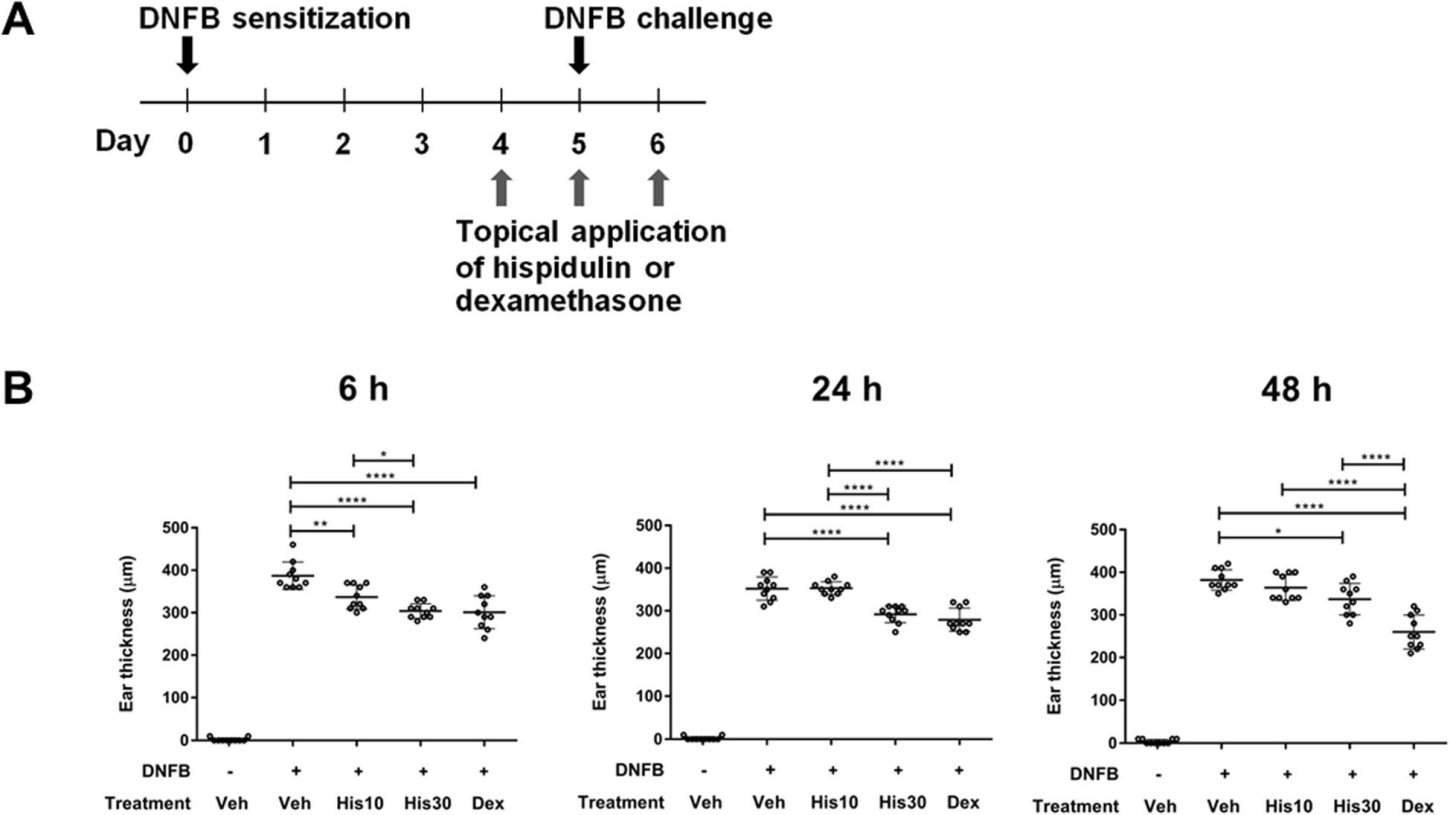
Attenuated CHS responses by hispidulin treatment. **a** The experimental design for induction of CHS as mentioned in Materials and Methods. Hispidulin at concentrations of either 10 or 30 μg/ear (His10 and His30) or 30 μg/ear dexamethasone (Dex) was applied to both ears once daily on days 4–6. Mice applied with vehicle (Veh) alone were used as a non-sensitized control. **b** Measurement of ear thickness. Thickness was measured using a Vernier caliper at 6-, 24-, and 48-h post-challenge. All values are presented as mean ± standard deviation (*n* = 5; *p < 0.05, **p < 0.01, ***p < 0.001)

To evaluate the immunosuppressive effect of hispidulin on ACD, a CHS mouse model was designed using DNFB for sensitization and elicitation (Fig. 1a). Data presented in Fig. 1b show that DNFB-mediated mice developed marked ear swelling when compared to sham immunized mice. Treatment with hispidulin at both the 10 and 30 μg concentrations was able to significantly reduce ear swelling within 6 h when compared to the sensitized control. The decrease in ear thickness after being treated with 30 μg/ear hispidulin was comparable to the decrease in ear thickness observed after treatment with dexamethasone at 6- and 24-h post-challenge. The observed suppression was then substantially decreased at 48-h post-challenge when compared to dexamethasone; however, the 30 μg hispidulin-treated ears still showed observably better suppression than the sensitized control.

### Hispidulin-induced attenuation of ear edema and spongiosis in CHS mice

**Fig. 2:**
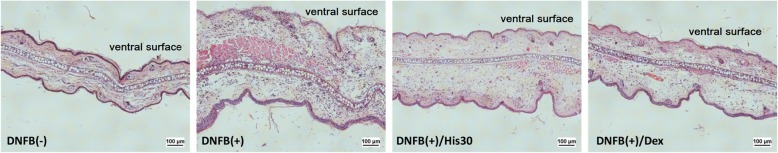
Representative photomicrographs of DNFB-sensitized mouse pinna after hispidulin treatment. Transverse sections of murine ears with no sensitization [DNFB (−)], DNFB sensitization [DNFB (+)], and DNFB sensitization together with treatment of 30 μg/ear hispidulin (His30) or 30 μg/ear dexamethasone (Dex) were compared at 24-h post-challenge. All tissues were stained with hematoxylin and eosin (H & E)

Histopathological changes in the excised ears revealed dermal thickness and infiltration of immune cells were both markedly increased in DNFB-sensitized mouse controls [Fig. 2, Additional file 1] when compared to the non-sensitized mice [DNFB (−)]. After hispidulin treatment of 30 μg/ear, ear edema and spongiosis were reduced to almost the same levels as those observed after dexamethasone application [DNFB (+)/His30 vs. DNFB (+)/Dex]. Remnants of infiltrated inflammatory cells was observed in the dermis in both the hispidulin and dexamethasone groups.

### Downregulation of IFN-γ gene expression in vivo after treatment with hispidulin

**Fig. 3:**
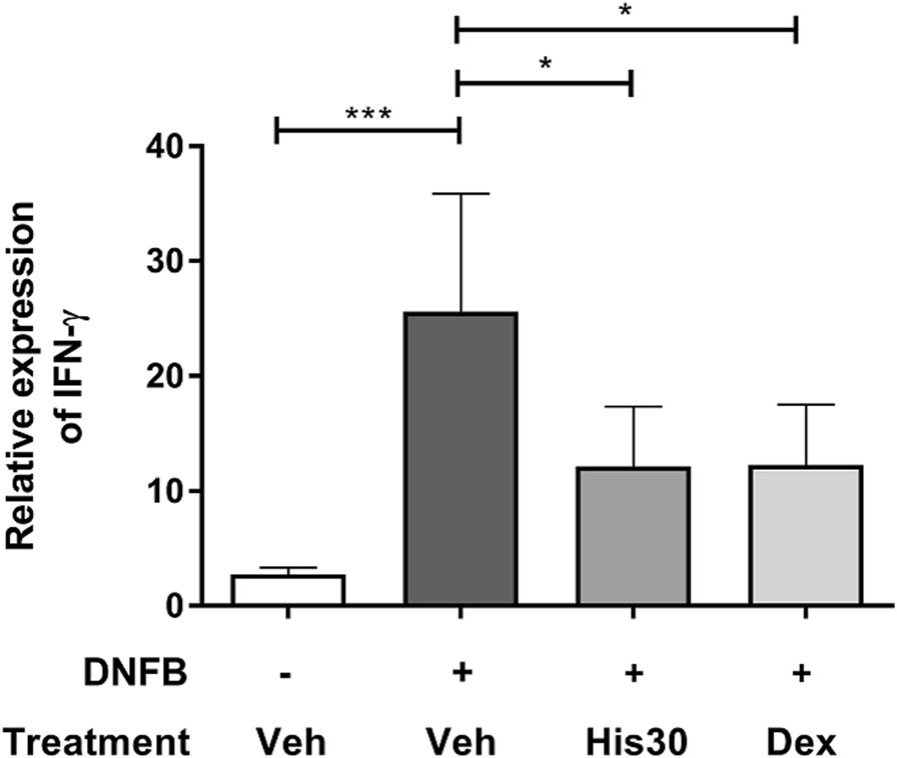
Relative mRNA expression of IFN-γ in mouse ears. Relative mRNA expression of IFN-γ to β-actin in ear tissues from mice with or without DNFB sensitization (+,-) and DNFB-sensitized mice with 30 μg/ear hispidulin (His30) or 30 μg/ear dexamethasone (Dex) or vehicle alone (sensitized control) were measured at 24-h post-challenge. All values are presented as mean ± standard deviation (*n* = 5; *p < 0.05, ***p < 0.001)

As Th1 cells secreting IFN-γ play an important role, the mRNA expression of IFN-γ in excised ear tissue was determined using real-time PCR. Relative mRNA expression of IFN-γ to that of β-actin was considerably increased (*p* = 0.0002) after DNFB sensitization and challenge when compared to that of the non-sensitized group (Fig. 3). Hispidulin treatment of 30 μg/ear was able to significantly downregulate IFN-γ expression (*p* = 0.0236), with results comparable to those observed after treatment with dexamethasone.

### Hispidulin-induced inhibition of T cell proliferation and Th1 cell differentiation in vitro

**Fig. 4:**
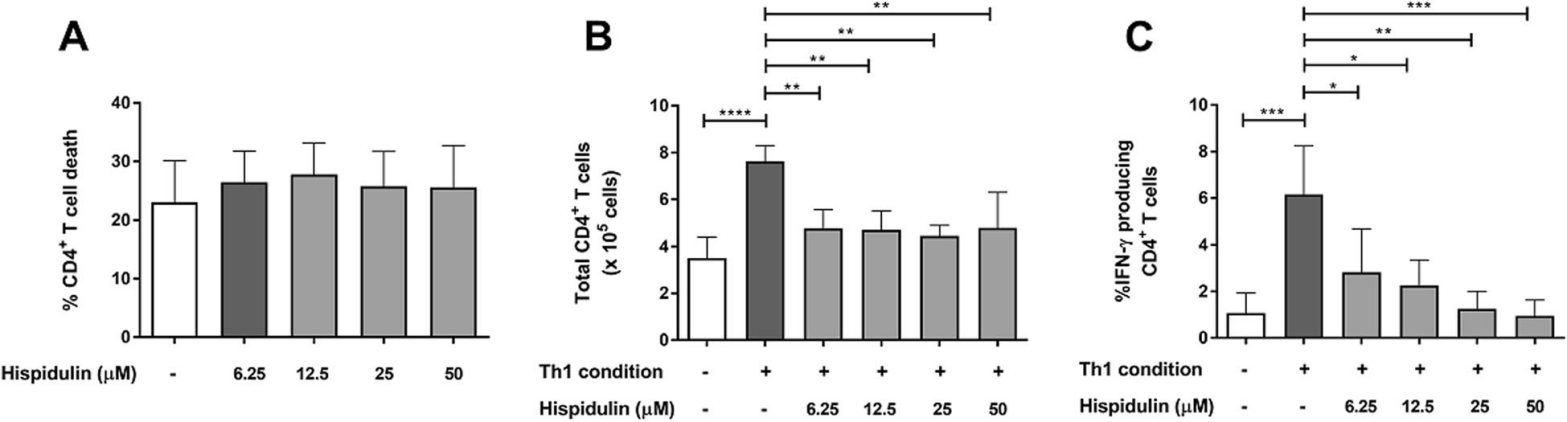
Effect of hispidulin on Th1 cell differentiation. **a** Cytotoxicity of hispidulin. Isolated naïve CD4^+^ T cells were cultured with hispidulin (0–50 μM) for 7 days. **b** and **c** Th1 cell differentiation in the presence of hispidulin. Isolated naïve CD4^+^ T cells were cultured under the Th1-skewing condition in the presence of hispidulin (0–50 μM). Total numbers of CD4^+^ T cells and percentages of IFN-γ producing CD4^+^ T cells were measured. All values are presented as mean ± standard deviation (*n* = 4; *p < 0.05, **p < 0.01, ***p < 0.001)

For drug cytotoxicity, the tests revealed no change in the percentages of CD4^+^ T cell death compared to untreated controls (Fig. 4a). The total number of CD4^+^ T cells and the percentage of IFN-γ-producing CD4^+^ T cells cultured in the Th1-skewing condition were significantly higher than the total number and percentage of cells cultured in complete medium (Fig. 4b and c; *p* < 0.0001 and *p* = 0.0007, respectively), which suggests that the Th1-skewing condition successfully induced Th1 cell differentiation. Hispidulin at all concentrations effectuated marked suppression of CD4^+^ T cell proliferation when compared to the suppression observed in the untreated control (*p* < 0.01), and no difference was observed among the 4 different hispidulin concentrations (Fig. 4b). However, hispidulin significantly reduced frequencies of IFN-γ-producing CD4^+^ T cells in a dose-dependent manner when compared to those observed in the untreated control (Fig. 4c; *p* < 0.01 for 6.25 and 12.5 μM, *p* = 0.0010 for 25 μM and *p* = 0.0005 for 50 μM). These findings suggest that hispidulin effectively inhibited CD4^+^ T cell function, most notably IFN-γ production, without interrupting cell frequencies.

## Discussion

In this study, we demonstrated the immunosuppressive effect of hispidulin on ACD using in vivo assay in CHS mouse model to simulate ACD reaction, and in in vitro study to demonstrate the effect of hispidulin on Th 1 cells. The animal models employed in this study showed that sensitization and elicitation with DNFB increased ear thickness, edema, and spongiosis, as well as increasing mRNA expression level of IFN-γ in inflamed tissues. These responses suggest the suitability of our CHS protocol.

We found that hispidulin at 10 and 30 μg/ear were able to reduce ear swelling within 6 h post-challenge, which confirms the previously reported findings that hispidulin inhibited 12-O-tetradecanoylphorbol-13-acetate (TPA)-induced murine ear edema [9, 10], and inhibited croton oil-induced dermatitis in mouse ears [12]. We also found a 30 μg/ear dose of hispidulin to have an inhibitory effect comparable to that of a 30 μg/ear dose of dexamethasone, which is similar to a dose causing 50% inhibition of inflammatory responses in vivo (ED_50_) at 0.3 μmol/cm^2^ (~ 25.5 μg/ear) in croton oil-induced mice [12]. This inhibitory dose was 7.4 times lower than the EC_50_ reported in TPA-induced mice treated with hispidulin isolated from a medicinal plant (223 μg/ear) [9]. It is, therefore, suggested that the effective doses from experiments using standard or purified compounds are more accurate and reliable than the effective doses from experiments using isolated compounds due to the absence of interference from other constituents in plant extracts.

Hispidulin was also reported to have good penetrative activity for topical delivery due to its structure and physicochemical properties, with percutaneous absorption within 3 h [7, 17]. Our results showed that hispidulin 10 μg/ear significantly suppressed ear swelling at 6-h post-challenge, and then its suppressive effect was reduced to almost the same level as the stimulated control at 24- and 48-h post-challenge. This may be due to the low concentration of hispidulin, which adversely affects its flux to the inflammation site over time. When the concentration was increased to 30 μg/ear, the amount of hispidulin as a reservoir was presumably sufficient to maintain the flux longer and to contribute to a greater amount of absorption, which resulted in better suppression that was comparable to dexamethasone at both 6- and 24-h post-challenge before the depletion of hispidulin at 48-h post-challenge. In overall comparison, dexamethasone was found to be more potent than hispidulin based on its continued efficacy at 48-h post-challenge, and the fact that it performed better relative to the prevention of enlargement of ear thickness, edema, and spongiosis in vivo.

Since Th1 lymphocytes play a pivotal role in ACD pathogenesis, the mRNA expression level of IFN-γ (which is upregulated in chronic ACD) in excised CHS murine pinna was measured to ensure the inhibitory effect of hispidulin at 30 μg/ear. Hispidulin was found to possess suppressive properties equivalent to those of dexamethasone by downregulating IFN-γ gene expression to the same level. Additionally, treatment with hispidulin at concentrations ranging from 6.25–50 μM significantly decreased the total numbers of CD4^+^ T cells, and a similar degree of inhibition was found among the different concentrations. However, dose-dependent suppression was observed for intracellular IFN-γ production, and hispidulin at a low dose of 6.25 μM was able to achieve only 50% inhibition. Hispidulin at all concentrations up to 50 μM was confirmed as safe by not inducing CD4^+^ T cell death. Taken together, these findings permit us to conclude that hispidulin regulated CHS reaction via suppression of Th1 cell function in IFN-γ production, and not via reduction in Th1 cell frequencies. Our findings are in contrast to those of another study that reported that hispidulin at concentrations of 1–10 μM significantly inhibited T cell proliferation in a dose-dependent manner [13]. However, that report did not identify which T cell subsets were suppressed by hispidulin, nor was any investigation of intracellular cytokine production described.

## Conclusions

This study demonstrates the immunosuppressive property of hispidulin in ACD via reduction of infiltrated immune cells at the inflammation site, downregulation of IFN-γ gene expression, and suppression of Th1 skewing reaction (i.e., IFN-γ production of CD4^+^ T cells), all of which result in decreased edema, spongiosis and ear swelling. Hispidulin also exhibits its inhibitory effect in a dose-dependent manner without inducing cell death and causing skin irritation. The results of this study suggest hispidulin as a novel immunosuppressant drug candidate for treatment of allergic contact dermatitis.

## Supplementary information

**Additional file 1. **Measurement of dermis thickness. Dermis thickness, excluding cartilage and epidermis, was measured by using photomicrographs of ears at 48-h post-challenge. All values are presented as mean ± standard deviation (*n* = 5; *p < 0.05, ***p < 0.001).

## Data Availability

All data generated or analyzed during this study are freely to any scientists wishing to use them for non-commercial purposes upon requested.

## Abbreviations

ACD: Allergic contact dermatitis
ANOVA: Analysis of variance
APC: Antigen presenting cells
BFA: Brefeldin A
CD: Cluster of differentiation
cDNA: Complementary deoxyribonucleic acid
CHS: Contact hypersensitivity
COX: Cyclooxygenase
Dex: Dexamethasone
DNCB: 2,4-dinitrochlorobenzene
DNFB: 1-fluoro-2,4-dinitrobenzene
FBS: Fetal bovine serum
FITC: Fluorescein isothiocyanate
H&E: Hematoxylin and Eosin
His: Hispidulin
HO: Heme oxygenase
ICS: Intracellular cytokine staining
IFN-γ: Interferon-gamma
IL: Interleukin
iNOS: Inducible nitric oxide synthase
JNK: c-Jun N-terminal kinase
LPS: Lipopolysaccharide
mL: Milliliter
NF-κB: Nuclear factor kappa-light-chain-enhancer of activated B cells
ng: Nanogram
NO: Nitric oxide
Nrf2: Nuclear factor erythroid 2-related factor 2
OX: Oxazone
PBS: Phosphate buffered saline
PCR: Polymerase chain reaction
PE: Phycoerythrin
PGE2: Prostaglandin E2
PI: Propidium iodide
PMA: phorbal 12-myristate 13-acetate
RNA: Ribonucleic acid
RPMI: Roswell Park Memorial Institute
Th: T helper
TNCB: 2,4,6-trinitrochlorobenzene
TPA: 12-O-tetradecanoylphorbol-13-acetate
U: Units
Veh: Vehicle
μg: Microgram
μM: Micromolar
