# Supplementary material for: Immunosuppressive effect of hispidulin in allergic contact dermatitis
Source: BMC Complement Altern Med. 2019 Oct 15;19:268. doi: 10.1186/s12906-019-2689-z (PMC6792202; doi:10.1186/s12906-019-2689-z)

Additional file 1. Measurement of dermis thickness. Dermis thickness, excluding cartilage and epidermis, was measured by using photomicrographs of ears at 48-hours post-challenge. All values are presented as mean ± standard deviation (n=5; *$p < 0.05$, ***$p < 0.001$).

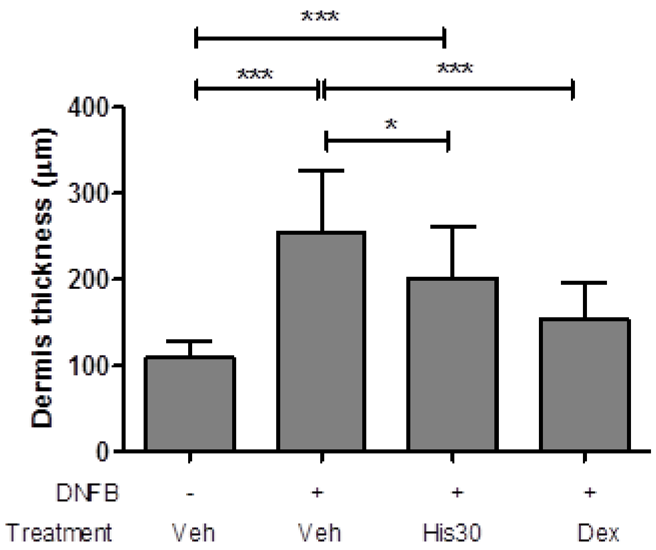

Supplement: Supplementary file 1 — Additional file 1. Measurement of dermis thickness. Dermis thickness, excluding cartilage and epidermis, was measured by using photomicrographs of ears at 48-h post-challenge. All values are presented as mean ± standard deviation (n = 5; *p < 0.05, ***p < 0.001). [file 12906_2019_2689_MOESM1_ESM.pdf]
